# Relationship Between Intraocular Pressure and Carbonic Anhydrase Enzyme Activity in Cattle: Implications for Veterinary Ophthalmology

**DOI:** 10.1002/vms3.70833

**Published:** 2026-02-20

**Authors:** Selvinaz Yakan, Cafer Tayer İşler, Mucip Genişel, Kader Yolcu, Ahmet Bayat, İlyas Aslan

**Affiliations:** ^1^ Animal Health Department Eleşkirt Celal Oruç School of Animal Production Ağrı İbrahim Çeçen University Ağrı Türkiye; ^2^ Faculty of Veterinary Medicine Department of Surgery Mustafa Kemal Üniversity Hatay Türkiye; ^3^ Faculty of Pharmacy, Department of Pharmaceutical Botany Ağrı İbrahim Çeçen University Ağrı Türkiye; ^4^ Department of Agricultural Business Eleşkirt Celal Oruç School of Animal Production, Ağrı İbrahim Çeçen University Ağrı Türkiye; ^5^ Department of Animal Science Institute of Graduate Programs Ağrı İbrahim Çeçen University Ağrı Türkiye

**Keywords:** Brown Swiss cows carbonic anhydrase, intraocular pressure in cattle, pregnancy, veterinary ophthalmology

## Abstract

This study evaluated the relationship between intraocular pressure (IOP) and carbonic anhydrase (CA) activity in healthy pregnant and non‐pregnant Brown Swiss cows. Fourteen cows (seven pregnant, seven non‐pregnant; average age 4.5 ± 0.3 years; body weight 500 ± 50 kg) were included. After clinical and ophthalmologic examinations, IOP was measured in both eyes using a rebound tonometer on 10 consecutive mornings (9–12 AM). Blood samples were collected for the determination of plasma CA activity. Data were analysed with parametric and non‐parametric tests, and right‐left eye agreement was assessed via Bland‐Altman analysis. Pregnant cows showed significantly lower mean IOP (22.19 ± 1.43 mmHg) than non‐pregnant cows (24.50 ± 1.60 mmHg; *p* < 0.001), with no significant difference between the eye sides. Plasma CA activity did not differ between groups, but IOP changes correlated positively with CA level changes in pregnant (*R* ^2^ = 0.31; *p* < 0.001) and non‐pregnant cows (*R* ^2^ = 0.38; *p* = 0.001). Pregnancy significantly reduces IOP in cattle, and CA activity is associated with IOP changes, suggesting that hormonal and hemodynamic alterations during pregnancy may influence intraocular fluid dynamics. The similarity between eyes facilitates clinical evaluation. The findings may inform further studies on the role of CA in ocular physiology during pregnancy.

## Graphical abstract

Schematic representation of the relationship between intraocular pressure (IOP) and carbonic anhydrase (CA) activity in pregnant and non‐pregnant cattle. Pregnancy is associated with a decrease in intraocular pressure, while plasma carbonic anhydrase activity shows a moderate positive correlation with IOP. These findings provide species‐specific insight into ocular physiology during pregnancy in cattle.

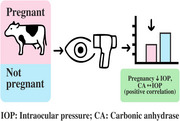

## 1 Introduction

Intraocular pressure (IOP) is a dynamic parameter that maintains the shape and optical function of the eyeball (Forrester et al. 2021; Cellat and İşler 2022; Levin et al. 2024). It is affected by the balance between aqueous humour production and outflow, and carbonic anhydrase (CA) enzymes play a crucial role in this process (Freddo et al. 2021). CA enzymes catalyse the secretion of bicarbonate ions from the aqueous humour, regulating fluid production and thereby modulating IOP (To et al. 2002). The use of CA inhibitors in human glaucoma treatment proves the role of these enzymes in regulating IOP (Supuran 2008). However, in the field of veterinary ophthalmology, the effects of physiological conditions such as pregnancy on IOP (Yakan and İşler 2021, 2022; Özcan et al. 2024; Yakan et al. 2024) and CA activity have so far been investigated in only a limited number of studies (Yakan et al. 2024).

Pregnancy is a complex process that causes metabolic, hormonal and hemodynamic changes in mammals (Schmidt‐Nielsen 1997). In human studies, increases in oestrogen and progesterone levels during pregnancy have been shown to influence ocular parameters, such as IOP (Ziai et al. 1994; Wang et al. 2017; Bujor et al. 2021). Low IOP, especially in pregnant women, has been linked to hormonal changes that affect aqueous humour dynamics (Green et al. 1988; Drake and Vajaranant 2016; Wang et al. 2017). Similarly, although the role of CA activity in placental pH regulation and fluid homeostasis during pregnancy has been studied (Ridderstråle et al. 1997; Rosen et al. 2001; Karahan et al. 2009), data on the relationship between ocular CA activity and pregnancy remain limited (Yakan et al. 2024).

IOP physiology in cattle differs from that in humans, resulting from interspecies variations in anatomical structure and fluid dynamics (Gum et al. 1998; Gelatt 2014; Levin et al. 2024). In cattle, IOP measurements are generally reported to be in the range of 18–28 mmHg (Gum et al. 1998; Andrade et al. 2011; Peche and Elue 2018; Yakan et al. 2021), but it is unclear how physiological conditions such as pregnancy affect this range (Yakan and İşler 2021, 2022; Yakan et al. 2024). Additionally, a comparative analysis of the systemic and ocular roles of CA enzymes in ruminants is essential due to the metabolic adaptations (Friedland and Maren 1984; Schmidt‐Nielsen 1997; Supuran 2008; García‐Llorca et al. 2024). The increased metabolic demand in the body during pregnancy, and the extensive physiological changes that occur, particularly in the eye (Pei and Li 2025), as well as changes in CA activity (Chegwidden 2021), suggest that ocular CA may be similarly modulated.

This study aimed to investigate the relationship between IOP and CA activity in cattle, to contribute to the understanding of the physiological basis for potential CA‐related mechanisms in veterinary medicine. We hypothesized that pregnancy lowers IOP and alters CA activity, leading to a positive correlation between CA and IOP levels in cattle.

## 2 Materials and Methods

### 2.1 Ethical Approval and Animal Welfare

The study was approved by the Animal Experiments Center Ethics Committee (HADMEK) of the Ministry of Agriculture and Forestry (Approval No. 2023/280.03, dated 3 March 2023). All procedures were conducted in accordance with Animal Protection Law No. 5199, the Regulation on the Working Procedures and Principles of Animal Experiments Ethics Committees, and ARRIVE guidelines. IOP measurements were performed using appropriate handling techniques to minimize stress to the cows. Blood samples were collected by a veterinarian under aseptic conditions and using proper vascular access techniques. The general health of the cows was monitored after the procedures, and no adverse effects or complications were observed throughout the study.

### 2.2 Animals

The study involved 14 healthy female Brown Swiss cows, including seven pregnant and seven non‐pregnant individuals, housed at the Celal Oruç Animal Production School Training, Research, and Application Farm of Ağrı İbrahim Çeçen University. Brown Swiss cows were selected for their calm temperament, large corneal surface and suitability for repeated IOP measurements. Moreover, cattle provide a relevant model for large domestic ruminants, allowing for a reliable assessment of ocular physiology (Gum et al. 1998; Gelatt 2014; Levin et al. 2024). The use of a single breed ensured homogeneity in physiological and handling characteristics, thereby minimizing variability in study outcomes. The mean age of the cows was 4.5 ± 0.3 years (range: 4–5 years), and the mean body weight was 500 ± 50 kg (range: 450–550 kg). All cows were maintained under uniform farm conditions (temperature: 18°C–22°C, humidity: 50%–60%), fed a standard ruminant diet and protected from environmental stressors. Housing conditions were carefully designed to ensure optimal health and stable physiological states. Daily care was provided by experienced personnel who closely monitored the cows for any signs of illness or stress. These measures improved internal validity and minimized environmental confounding factors (Hubrecht and Kirkwood 2010).

### 2.3 Pregnancy Examination

Pregnant cows were selected from the late gestation period (approximately 250 ± 10 days of pregnancy), corresponding to the last third of gestation (Days 181–280) when the physiological effects of pregnancy are most pronounced (Arthur 2001). The gestational stage and pregnancy status were determined by rectal palpation and confirmed using farm breeding records and periodic ultrasonographic examinations performed with a portable transrectal ultrasound device (Hasvet Veterinary Ultrasound Scanner, Türkiye) equipped with a convex probe operating at 3.5–5 MHz. This stage is characterized by high oestrogen and progesterone levels and increased cardiovascular and metabolic demands, during which ocular physiological changes are most evident. During the clinical examination, body weight, age and physiological parameters (body temperature, heart rate and respiratory rate) were recorded for each cow to assess overall health status and facilitate further statistical evaluation.

### 2.4 Intraocular Pressure Measurement

Only clinically healthy cows were included in the study. IOP measurements were performed using a rebound tonometer (TonoVet, iCare Finland Oy, Vantaa, Finland). Measurements were taken once daily for 10 consecutive days, immediately after morning feeding, between 9:00 AM and 12:00 PM. Before each session, the probe base (device housing) was inspected and cleaned according to the manufacturer's guidelines, while single‐use disposable probe tips were replaced for each measurement session and were not reused. Since no cattle‐specific validated calibration mode is available, IOP measurements were performed using the standard setting of the rebound tonometer, and no topical anaesthesia was required due to the non‐invasive nature of the device. During IOP measurements, each cow was gently restrained in a standing position by an experienced assistant to minimize stress and prevent head movement. Care was taken to avoid applying excessive pressure on the neck or thorax, ensuring that the restraint did not affect ocular blood flow or IOP readings. This handling method allowed accurate tonometric measurements while maintaining animal welfare. For each eye, the device automatically performed six consecutive measurements within a single session, providing the mean IOP value, which was recorded in millimetres of mercury (mmHg). Disposable probe tips were used to prevent corneal trauma. Cows showing any ocular or systemic abnormalities were excluded. Randomization was not applicable since all cows were evaluated under the same standardized conditions. Full blinding was not applied; however, measurement reliability was ensured by following standardized protocols and performing all measurements under consistent conditions by the same experienced researcher. The 10‐day study period proceeded according to the established protocol, and no deviations, complications or adverse events were encountered during this time.

### 2.5 Assessment of Carbonic Anhydrase Activity

Blood samples were taken after the morning feeding (at 9–12 AM) simultaneously with IOP measurements and were collected in EDTA tubes. They were stored at −80°C and then analysed. CA activity was measured Shimadzu UV‐1800 spectrophotometer (Shimadzu, Kyoto, Japan) in blood plasma samples. P‐nitrophenyl acetate (pNPA) was used as a substrate for esterase activity determination. In this method, the absorbance of p‐nitrophenol, formed by carbonic anhydrase‐catalysed pNPA hydrolysis, was measured at 348 nm in quartz cuvettes at 25°C, and activity values were calculated using the molar absorptivity coefficient (*ε*_348_ = 5 × 10^3^ M^−1^ cm^−1^) (Verpoorte et al. 1967).

### 2.6 Statistical Analysis

All statistical analyses were performed using GraphPad Prism (version 9.5.1) and MedCalc Statistical Software (version 22.009). Data normality was assessed with the Shapiro–Wilk test. For normally distributed data, independent samples t‐tests were used to compare group means; for non‐normally distributed data, the Mann–Whitney U test was applied. Paired samples t‐tests were used to assess differences between right (R‐IOP) and left (L‐IOP) eye measurements within each group. Agreement between measurement methods was evaluated using Bland–Altman analysis (Bland and Altman 1986), reporting bias, upper (ULA) and lower (LLA) limits of agreement. Effect size was expressed as Cohen's *d* with 95% confidence intervals (Cohen 1988). Point‐biserial correlation analysis was performed to evaluate the relationship between pregnancy status and body weight. Linear regression analysis was used to examine associations between changes in IOP and CA activity within groups. Variables that showed significant associations in univariate analyses were further analysed using multivariate regression models, adjusting for potential confounders, including age, body weight and pregnancy status. Data analyses were also conducted in Python (version 3.10) using the SciPy, Statsmodels and Matplotlib libraries. A *p* value < 0.05 was considered statistically significant. After completion of all analyses, a post‐hoc power analysis was performed using G*Power 3.1, based on the observed effect size (Cohen's *d* = 1.49). The analysis suggested that the study sample size provided acceptable statistical power (approximately 0.91) to detect between‐group differences in IOP at a 0.05 significance level. Although the total number of animals was limited (*n* = 14), each cow underwent repeated IOP measurements for 10 consecutive days, resulting in a total of 140 data points (70 per group). This repeated‐measurement design substantially increased the statistical power and reliability of the results by reducing within‐subject variability and measurement error.

Similar experimental designs with comparable or even smaller sample sizes have been reported in veterinary ophthalmology, particularly in studies focusing on physiological parameters under controlled conditions. Furthermore, the homogeneity of the animals (same breed, age range and housing conditions) minimised confounding factors and strengthened the internal validity of the findings. Therefore, despite the relatively small number of animals, the repeated‐measurement protocol and strict control of experimental conditions provided sufficient analytical robustness and statistical reliability for the study objectives.

## 3 Results

A total of 14 healthy Brown Swiss cows participated in the study, with seven classified as pregnant and seven as non‐pregnant. A statistically significant difference in body weight was observed between the two groups: pregnant cows (515.7 ± 28.3 kg) were heavier than non‐pregnant cows (455.7 ± 33.9 kg) (*p* = 0.0055). The effect size (Cohen's *d*) for body weight was 1.92 (95% CI, 0.48–3.36). Point‐biserial correlation analysis also showed a positive relationship between pregnancy status and body weight (*R* = 0.70, *p* < 0.01), indicating that pregnancy had a significant influence on body weight.

The mean plasma CA activity was 0.13 ± 0.01 U/mg in pregnant cows and 0.14 ± 0.02 U/mg in non‐pregnant cows, with no statistically significant difference between the groups (*p* = 0.40). No differences were found between the groups in terms of age or CA activity (*p* > 0.05). Demographic and clinical characteristics of both groups are summarized in Table 1.

**TABLE 1:** Demographic and clinical variables of pregnant and non‐pregnant cows.

Variable	Pregnant group (*n* = 7)	Non‐pregnant group (*n* = 7)	*p* value
Age (years, mean ± SD)	4.52 ± 0.19	4.56 ± 0.19	0.73^a^
Weight (kg, mean ± SD)	515.7 ± 28.3	455.7 ± 33.9	0.006^a^
CA activity (U/mg, mean ± SD)	0.13 ± 0.01	0.14 ± 0.02	0.40^a^

Abbreviation: CA, carbonic anhydrase; SD, standard deviation.

^a^ Independent samples *t*‐test.

### 3.1 Comparison of Right and Left Eye Intraocular Pressure

**FIGURE 1:**
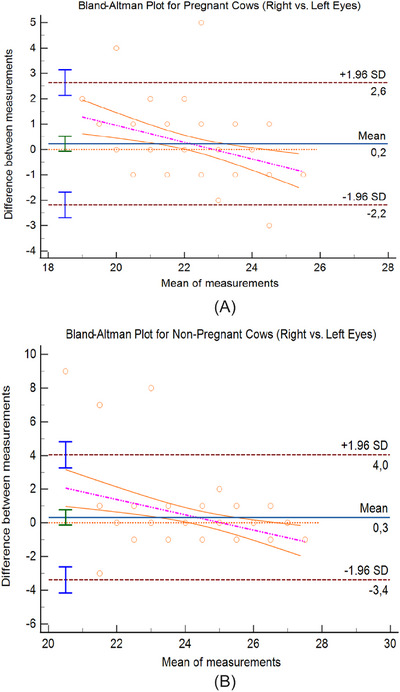
(A) Bland–Altman plot for pregnant cows (right vs. left eyes). Bland–Altman plot illustrating the agreement between right and left intraocular pressure (IOP) measurements in pregnant cows. The solid blue line represents the mean difference (bias) between the two eyes (0.2 mmHg). The dashed brown lines indicate the upper and lower limits of agreement (± 1.96 SD; + 2.6 mmHg and – 2.2 mmHg, respectively). The orange line shows the fitted regression trend between measurement differences and mean IOP, while the magenta dashed line denotes the 95% confidence interval of the regression. Each orange circle represents an individual measurement. The results demonstrate a high degree of agreement, indicating that right and left eye IOP values can be used interchangeably in pregnant cows. (B) Bland–Altman plot for non‐pregnant cows (right vs. left eyes). Bland–Altman plot illustrating the agreement between right and left intraocular pressure (IOP) measurements in non‐pregnant cows. The solid blue line represents the mean difference (bias) between the two eyes (0.3 mmHg). The dashed brown lines indicate the upper and lower limits of agreement (± 1.96 SD; + 4.0 mmHg and – 3.4 mmHg, respectively). The orange line shows the fitted regression trend between measurement differences and mean IOP, while the magenta dashed line denotes the 95% confidence interval of the regression. Each orange circle represents an individual measurement. The results demonstrate strong agreement, confirming that right and left eye IOP values can be used interchangeably in non‐pregnant cows.

In pregnant cows, the mean right‐eye IOP (R‐IOP) was 22.39 ± 1.37 mmHg, and the mean L‐IOP was 22.20 ± 1.80 mmHg. The difference was not statistically significant (*t*(6) = 1.02, *p* = 0.31; Cohen's *d* = 0.19, 95% CI, −0.18 to 0.57). Similarly, in non‐pregnant cows, R‐IOP averaged 24.50 ± 1.52 mmHg, and L‐IOP averaged 24.17 ± 2.16 mmHg, with no significant difference (*t*(6) = 1.45, *p* = 0.15; Cohen's *d* = 0.17, 95% CI, −0.12 to 0.46). Bland–Altman analysis confirmed high agreement between R‐ and L‐eye measurements. In pregnant cows, the bias between R and L eyes was 0.23 mmHg, with an ULA of + 2.64 mmHg and a LLA of −2.18 mmHg (Figure 1A). In non‐pregnant cows, the bias was 0.33 mmHg, with ULA = + 4.04 mmHg and LLA = − 3.38 mmHg (Figure 1B). The narrow limits and minimal bias suggest that R and L eye measurements were interchangeable within both groups.

### 3.2 Effect of Pregnancy on Intraocular Pressure

**FIGURE 2:**
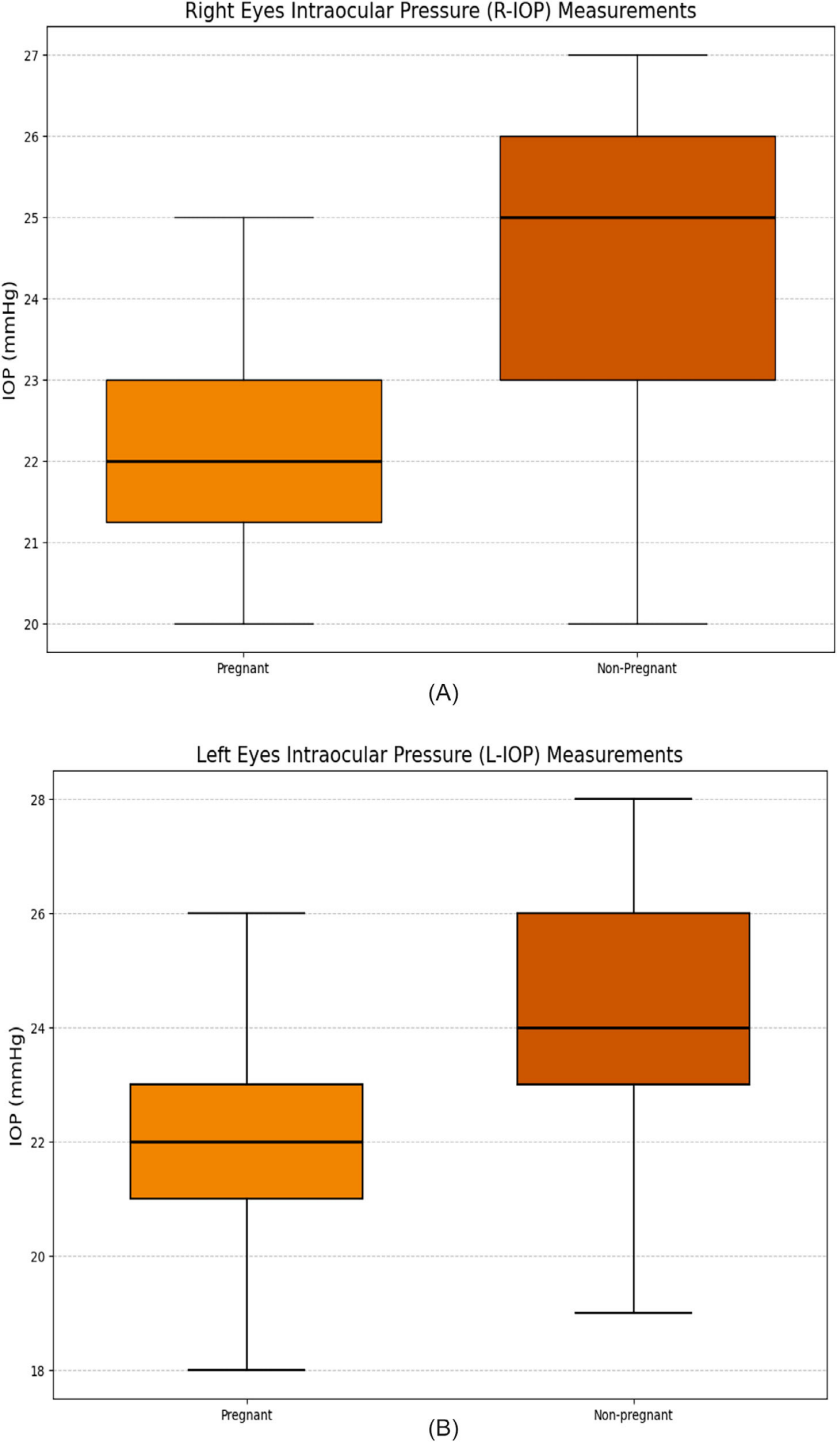
(A) Box plot showing the intraocular pressure (IOP, mmHg) values measured in the right eyes (R‐IOP) of pregnant and non‐pregnant cows. Each box represents the interquartile range (IQR), with the horizontal line inside indicating the median value. The whiskers denote the minimum and maximum values recorded over 10 consecutive days. The plot illustrates that IOP was significantly lower in pregnant cows compared to non‐pregnant cows (*p *< 0.001). (B) Box plot showing the intraocular pressure (IOP, mmHg) values measured in the left eyes (L‐IOP) of pregnant and non‐pregnant cows. Each box represents the interquartile range (IQR), with the horizontal line inside indicating the median value. The whiskers denote the minimum and maximum IOP values recorded over 10 consecutive days. The data show that IOP was significantly lower in pregnant cows compared to non‐pregnant cows (*p *< 0.001).

To assess the impact of pregnancy on IOP, both R‐IOP and L‐IOP values were compared between groups. Measurements were taken over 10 consecutive days, and daily averages were calculated for each cow. Pregnant cows showed significantly lower IOP values than non‐pregnant cows. The mean R‐IOP was 22.39 ± 1.37 mmHg in pregnant cows versus 24.50 ± 1.52 mmHg in non‐pregnant cows (*p *< 0.0001; Cohen's *d* = −1.54, 95% CI, −1.85 to −1.22). Similarly, the mean L‐IOP was 22.20 ± 1.80 mmHg in pregnant cows and 24.17 ± 2.16 mmHg in non‐pregnant cows (*p* < 0.0001; Cohen's *d* = −1.47, 95% CI, −1.85 to −1.10). The overall mean IOP of pregnant cows was 22.19 ± 1.43 mmHg, significantly lower than that of non‐pregnant cows (24.50 ± 1.60 mmHg, *p* = 0.00012; Cohen's *d* = −1.49, 95% CI, −1.90 to −1.10). Mean and range values for both groups are illustrated in Figure 2A–B.

These findings indicate that pregnancy exerts a significant lowering effect on IOP in cattle.

### 3.3 Relationship Between Intraocular Pressure and Carbonic Anhydrase Activity

**FIGURE 3:**
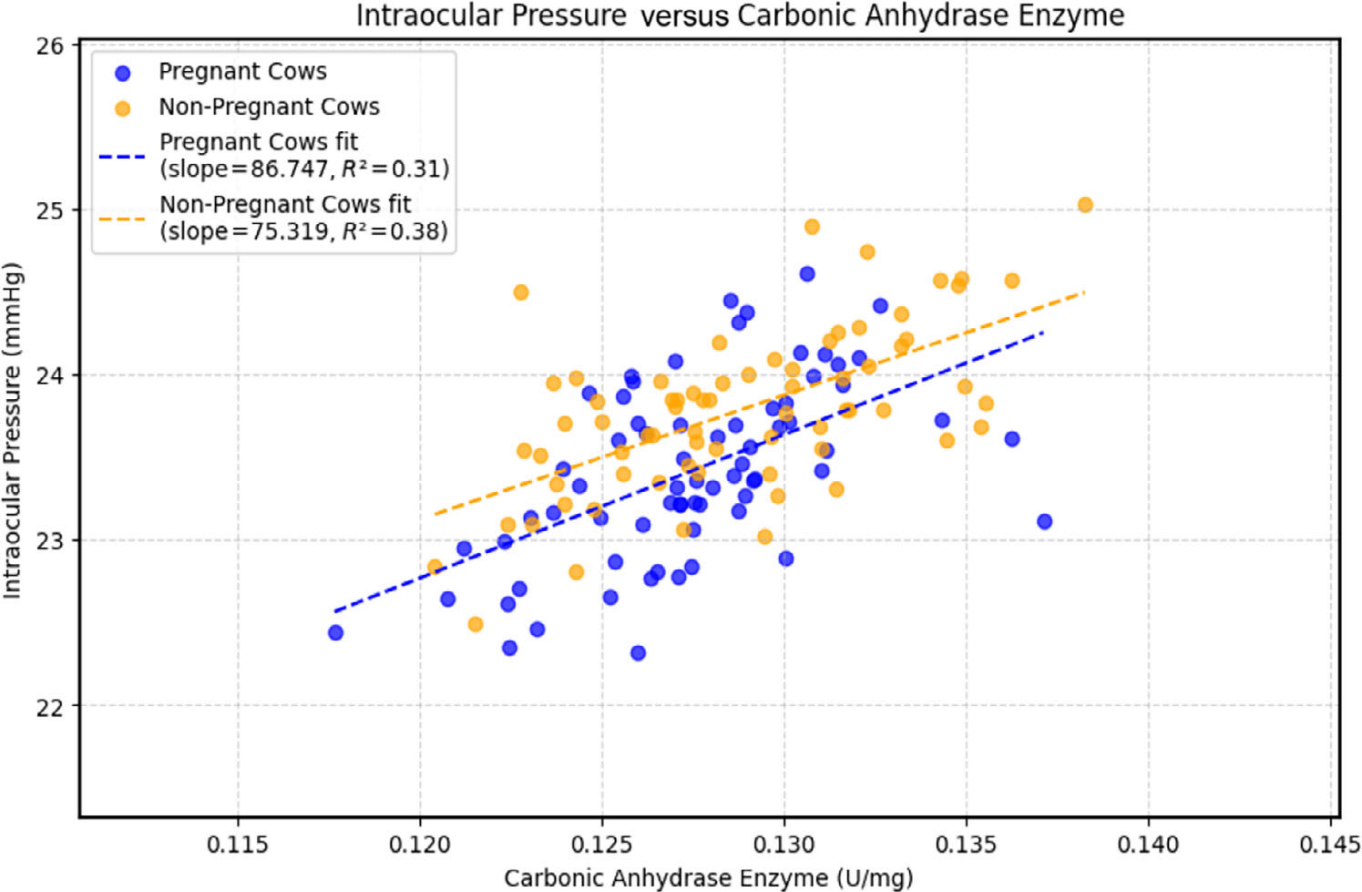
Scatter plot illustrating the relationship between intraocular pressure (IOP) and carbonic anhydrase (CA) enzyme activity in pregnant and non‐pregnant cows. Each point represents an individual measurement. The dashed blue line shows the regression fit for pregnant cows (slope = 86.747, *R* ^2^ = 0.31), and the dashed orange line represents the regression fit for non‐pregnant cows (slope = 75.319, *R* ^2^ = 0.38). The plot demonstrates a moderate positive correlation between CA activity and IOP in both groups.

Univariate linear regression analysis revealed a moderate positive association between CA activity and IOP in both groups. In pregnant cows, the regression slope was 86.75 (*R* ^2^ = 0.31, *p* < 0.001), and in non‐pregnant cows, it was 75.32 (*R* ^2^ = 0.38, *p* = 0.001). Residual plots showed random dispersion around zero without heteroscedasticity, confirming model validity. The relationship between IOP and CA activity changes in both groups is depicted in Figure 3. In the multivariate linear regression model, the association between CA level change and IOP remained significant (*β* = 0.82, SE = 0.11, *p* < 0.001) even after adjusting for age, body weight and pregnancy status. None of these covariates showed an independent association with IOP (all *p *> 0.05). The model showed a good overall fit (*R* ^2^ = 0.42, adjusted *R* ^2^ = 0.36), suggesting that variation in CA activity was the primary predictor of IOP change after adjustment.

## 4 Discussion

IOP is regulated by multiple systemic and ocular factors, one of which is pregnancy (Murgatroyd and Bembridge 2008; Yakan 2021). Pregnancy represents a complex physiological state characterised by extensive metabolic, hormonal and hemodynamic adaptations (Zakaria et al. 2022). During this period, elevations in progesterone and oestrogen levels can influence ocular tissues and alter aqueous humour dynamics (Wang et al. 2017; Kılavuzoğlu et al. 2018; Bujor et al. 2021). The reported effects of pregnancy on IOP vary considerably across studies, with some showing no significant change, while others report either an increase or a decrease (Naderan 2018; Anton et al. 2021; Zakaria et al. 2022; Pei and Li 2025). In feline models, Ofri et al. (2002) observed that IOP varied according to circulating progesterone levels. In contrast, Özcan et al. (2024) reported lower IOP values in pregnant cats compared to non‐pregnant ones, possibly due to hormonally mediated changes in ocular physiology. Human studies generally support the IOP‐lowering effect of progesterone, with reductions of approximately 2–3 mmHg, up to 10% during late gestation. The mechanism is proposed to involve enhanced aqueous humour outflow and reduced episcleral venous pressure associated with increased oestrogen, progesterone and relaxin concentrations. These physiological changes are transient, and IOP typically returns to pre‐pregnancy levels within a few months after parturition (Naderan 2018; Zakaria et al. 2022; Pei and Li 2025).

In the present study, IOP values were significantly lower in pregnant Brown Swiss cows compared with non‐pregnant cows (*p* < 0.001). This finding is consistent with reports in humans and other mammalian species that describe a reduction in IOP during pregnancy (Qureshi et al. 1996; Efe et al. 2012; Özcan et al. 2024). The observed decrease in IOP likely reflects the influence of pregnancy‐associated hormonal and physiological adaptations on intraocular fluid regulation.

Although CA enzyme activity is known to play an essential role in pH regulation and fluid homeostasis during pregnancy (Ridderstråle et al. 1997; Rosen et al. 2001; Karahan et al. 2009), its specific relationship with ocular physiology during gestation remains insufficiently defined. In the present study, plasma CA activity did not differ significantly between pregnant and non‐pregnant cows, suggesting that systemic and ocular CA regulation may function independently. Since plasma measurements alone may not accurately represent intraocular biochemical processes, future research should focus on direct assessments of CA activity in aqueous humour or other ocular fluids to better clarify its physiological role. Hormonal factors such as oestrogen and progesterone are likely to influence CA activity during pregnancy; however, this interaction was not directly demonstrated in the current study. Further investigations evaluating ocular CA expression under varying hormonal conditions would provide valuable mechanistic insight.

The observed positive association between CA activity and IOP was moderate but consistent across both groups, indicating that the enzyme may play an important role in IOP regulation, independent of the physiological alterations that occur during pregnancy. From a clinical perspective, considering pregnancy status in tonometric evaluations may improve diagnostic accuracy.

The similarity between right and left eye IOP measurements observed in this study is consistent with previous reports, indicating that both eyes can be used interchangeably in tonometric evaluations. This supports the reliability and reproducibility of IOP measurements in cattle. The Bland–Altman analysis further confirmed this consistency (Gum et al. 1998; Yakan et al. 2021; Özcan et al. 2024).

The significantly higher body weight observed in pregnant cows (*p* = 0.0055) likely reflects the increased metabolic and circulatory demands of pregnancy (Abrams et al. 2000; Dodd et al. 2024). These systemic changes may also influence intraocular fluid dynamics, warranting further investigation.

Overall, the present study demonstrates that pregnancy is associated with decreased IOP in cattle and a moderate positive correlation between IOP and CA activity. These results underscore the complex interaction between hormonal, metabolic and ocular parameters during pregnancy and emphasize the value of monitoring ocular changes in pregnant animals.

Bland–Altman analysis was performed using daily averaged IOP values to minimize intra‐animal variation and to increase data reliability given the limited number of animals. Although repeated measurements were obtained from the same individuals, this design was intended to enhance statistical robustness while maintaining measurement independence across days.

By providing quantitative data on plasma CA activity and its relationship with IOP, this study contributes species‐specific insights to the limited body of veterinary ophthalmic literature. The findings are primarily confirmatory in nature, filling a critical knowledge gap since no previous bovine studies have quantitatively linked plasma CA activity with IOP, particularly in relation to pregnancy. Although the present study demonstrates a moderate positive correlation between plasma CA activity and IOP, this relationship is correlational rather than mechanistic. The enzyme may play a role in regulating IOP through pathways influenced by pregnancy‐related hormonal changes. Further studies are required to clarify the causal mechanisms underlying this association.

This study has several limitations; the sample size was relatively small, which may limit the generalizability of the findings. Only plasma CA activity was measured, which may not fully represent ocular CA activity. All animals were of a single breed (Brown Swiss), and the short 10‐day measurement period may not capture long‐term physiological variations. Although body weight was included as a covariate in the regression model, potential residual confounding cannot be completely excluded. Future studies with larger populations, multiple breeds and direct measurements of ocular CA activity may build upon these findings and provide a broader understanding of the relationship between pregnancy and IOP in cattle.

In addition, this study focused exclusively on cows in late gestation, when pregnancy‐related hormonal and physiological changes are most pronounced. While this approach allowed clearer detection of pregnancy‐associated effects on IOP, it does not capture potential variations across different stages of gestation. Future longitudinal studies incorporating early, mid and late pregnancy time points, together with direct hormonal measurements, may provide a more detailed understanding of the temporal dynamics linking pregnancy, CA activity and IOP in cattle.

## 5 Conclusion

This study demonstrates that pregnancy lowers IOP in cattle and that CA activity shows a moderate positive association with IOP. These findings highlight the importance of considering physiological states such as pregnancy when interpreting IOP values in cattle. Furthermore, understanding the modulation of CA activity may provide new insights into ocular physiology and serve as a basis for future studies in veterinary ophthalmology.

## Author Contributions

**Selvinaz Yakan**: conceptualization, methodology, intraocular pressure measurements, data collection, formal analysis, writing – original draft, supervision. **Cafer Tayer İşler**: data curation, writing – review and editing. **Mucip Genişel**: carbonic anhydrase enzyme analysis, writing – review and editing. **Kader Yolcu**: pregnancy examination, blood sampling, writing – review and editing. **Ahmet Bayat**: intraocular pressure measurements, writing – review and editing. **İlyas Aslan**: intraocular pressure measurements, writing – review and editing. All authors checked the final version of the manuscript and reached a consensus.

## Funding

The authors have nothing to report.

## Ethics Statement

This study was reported to the Animal Experiments Center Ethics Committee (HADMEK) of the Ministry of Agriculture and Forestry with a letter dated 3 March 2023, and numbered 2023/280.03.

## Conflicts of Interest

The authors declare no conflicts of interest.

## Data Availability

The article data are available from the corresponding author upon request.
